# AAV microdystrophin gene replacement therapy for Duchenne muscular dystrophy: progress and prospects

**DOI:** 10.1038/s41434-025-00561-6

**Published:** 2025-08-15

**Authors:** Katarzyna Chwalenia, Vivi-Yun Feng, Nicole Hemmer, Hans J. Friedrichsen, Ioulia Vorobieva, Matthew J. A. Wood, Thomas C. Roberts

**Affiliations:** 1 Department of Paediatrics, University of Oxford, IDRM, Oxford, OX3 7TY UK; 2 Freie Universität Berlin, Kaiserswerther Str. 16-18, 14195 Berlin, Germany; 3 Eberhard-Karls-Universität Tübingen, Geschwister-Scholl-Platz, 72074 Tübingen, Germany; 4 MDUK Oxford Neuromuscular Centre, South Parks Road, Oxford, UK

**Keywords:** Drug delivery, Diseases, Gene expression

## Abstract

Duchenne muscular dystrophy (DMD) is caused by pathogenic sequence variants occurring in the *DMD* gene which lead to the loss of the dystrophin protein, a molecular ‘shock absorber’ that protects muscle from contraction-induced injury. The large size of the dystrophin open reading frame precludes delivery of the full-length protein using a single adeno-associated virus (AAV) vector, which led to the development of internally-deleted dystrophin minigenes encoding partially-functional dystrophin. Indeed, five such microdystrophin therapies have been assessed in various clinical programmes. In 2023, Elevidys (Sarepta Therapeutics) received accelerated approval based on levels of dystrophin as a surrogate biomarker. In 2024, it received full approval despite unclear efficacy (i.e. not meeting primary or secondary outcomes in a phase 3 trial). Additionally, in 2025, two DMD individuals treated with Elevidys died after acute liver failure. A separate microdystrophin therapy, PF-06939926 (Pfizer) was discontinued for both efficacy and safety reasons (including the deaths of two clinical trial participants). Solid Biosciences, Genethon, REGENXBIO, and Insmed continue to develop microdystrophin therapies differing in transgene structure, promoter sequences, and AAV serotype. Here we describe recent progress in AAV-microdystrophin therapeutics development, and discuss the challenges facing such approaches, including pre-existing anti-capsid immunity, anti-transgene immunity, the unknown functionality of microdystrophin transgenes, transduction of muscle stem cells, and long-term transgene persistence.

## Duchenne muscular dystrophy

Duchenne muscular dystrophy (DMD) is a severe, progressive muscle-wasting disorder caused by genetic loss of the dystrophin protein. This X-linked recessive neuromuscular disease is one of the most common inherited myopathies, affecting around 1 in 5000 live male births. Clinical manifestations (e.g. frequent falls, difficulties in climbing stairs, inability to run, and Gower’s sign) typically manifest at 2-3 years of age [1, 2]. The absence of dystrophin sensitizes the sarcolemma to contractile damage [3] leading to myonecrosis, which affects the majority of the skeletal musculature (including diaphragm) and the heart. Skeletal muscle tissue exhibits an exceptional capacity for regeneration. However, in DMD, healthy muscle fibers are progressively replaced by fat and fibrotic tissue, which decreases the ability of muscle tissues to regenerate and impairs the generation of force required for daily activities [4, 5]. DMD-affected boys typically become wheel-chair dependent by 10–12 years of age [6], with death usually occurring within the first three decades of life as a consequence of dilated cardiomyopathy, or weakening of the muscles that support breathing [7].

## The *DMD* gene

The gene that encodes the dystrophin protein (*DMD*) is unusually large, spanning ~2.2 Mb at Xp21. The transcript encoding the 427 kDa muscle isoform (Dp427m) (NM_004006/ENST00000357033) consists of 79 exons, with ~99% of the gene being intronic sequence. Transcription of this enormous gene takes ~10–16 h [8, 9] and produces a 14 kb mature mRNA with a ~ 11 kb open reading frame (ORF) [10]. The *DMD* locus also gives rise to multiple other dystrophin protein isoforms (as a consequence of distinct promoters, alternative splicing, and differential polyadenylation site usage), which exhibit distinct patterns of tissue expression [6].

Due to its large size, and the presence of multiple semi-redundant repeat domain sequences, the dystrophin gene is highly susceptible to de novo variants [6, 11]. Variants that introduce premature termination codons or disrupt the *DMD* reading frame lead to loss of functional dystrophin protein. The most common pathogenic variants are intragenic deletions, often spanning one or more exons and accounting for ~65% of all DMD cases [12]. Single nucleotide variants, duplications [13], and other small mutations make up the remainder of disease causing variants. Although pathogenic variants occur throughout the entire length of the gene, there are two deletion hotspots in regions spanning exons 3–9 and exons 45–55 [12, 14].

## The dystrophin protein

The Dp427m dystrophin protein isoform is a long rod-like protein that is localized to the cytoplasmic surface of the sarcolemma and acts as an organizing center for the DAPC (dystrophin-associated glycoprotein complex), which includes the dystroglycans (α and β), sarcoglycan complex, α-dystrobrevin, neuronal nitric oxide synthase (nNOS), sarcospan, syntrophins, syncoilin, and others [15, 16]. The dystrophin protein itself consists of four domains: (i) the N-terminal domain (NT), (ii) the central rod domain, (iii) the cysteine-rich domain, and (iv) the C-terminal domain (CT). The NT domain is encoded by exons 1–8 and comprises two calponin homology (CH) motifs that form the actin binding domain (ABD) which connects dystrophin with filamentous actin [6]. The central rod domain, encoded by exons 8–64 constitutes the largest part of the dystrophin protein, which provides flexibility and gives rise to its characteristic elongated rod shape [17]. It consists of 24 homologous spectrin-like repeats (R), with each repeat containing ~109 amino acids [18]. R11-15 comprise the second ABD, providing an additional connection between dystrophin and cytoskeletal actin [19]. Additional cytoskeleton contacts occur via interaction of repeats R20-23 with microtubules [20, 21]. Moreover, repeats R16-17 directly anchor nNOS to the sarcolemma [22, 23]. The spectrin-like repeats of the rod domain are interspersed by 4 proline-rich hinges (H), that contribute to the inherent elasticity of the dystrophin protein [24]. The 150 amino acid long cysteine-rich (CR) domain, encoded by exons 64–70 [6], is crucial for anchoring dystrophin to the sarcolemma through direct binding to β-dystroglycan [25]. Lastly, the CT domain is encoded by exons 71-79 [6], and contains binding sites for dystrobrevins and syntrophins [26, 27]. As such, dystrophin forms a mechanical link between myofiber cytoskeletal actin, via direct interactions which occur towards its N-terminus, and the extracellular matrix, via interaction with β-dystroglycan [28], which itself binds to α-dystroglycan and laminins [29], at its C-terminus. The dystrophin protein consists of multiple independent membrane-binding regions located in the R1-3, R10-12, CR, and CT regions [30]. Dystrophin also plays important non-mechanical roles in terms of scaffolding and cell signaling associated with numerous pathways due to its association with MEK, ERK, and nNOS [31, 32].

While some structural proteins such as utrophin (UTRN, a paralogue of dystrophin) have been found to be upregulated to partly compensate for the absence of dystrophin [33–35], the majority of DAPC members are significantly downregulated in dystrophic tissue [36–38], which contributes to mechanical damage and fiber necrosis in DMD patients [2]. Indeed, genetic loss of many of the other DAPC proteins is causative in multiple other muscular dystrophies [39].

## Becker muscular dystrophy

Pathogenic variants in the *DMD* gene can also cause the less severe allelic dystrophinopathy, Becker muscular dystrophy (BMD) [40]. In this case, the causative genetic lesion often does not disrupt the translation reading frame, but instead leads to the production of a partially-functional, internally-deleted dystrophin protein that retains its terminal protein binding partner capabilities. BMD has an incidence of ~1 in 10,000–100,000 live male births [41] and typically presents later in life relative to DMD [42]. The severity varies widely between individuals and ranges from effectively asymptomatic [43, 44] to DMD-like phenotypes [45]. BMD patients typically maintain their ability to walk until their third decade of life [46]. Generally, deletions at the 5ʹ end of the *DMD* gene are associated with an early-onset and more severe disease due to the loss of actin-binding properties, suggesting that the position of the variant plays an important role in determining disease severity [45]. However, a study of a BMD-affected family revealed that even closely-related individuals with the same variant can present with different severities of the disease, suggesting that other factors (e.g. genetic modifiers) might influence disease outcomes [47, 48]. Importantly, the size of the deletion does not necessarily correlate with the severity of the disease. Deletions leading to loss of up to 46% of the gene have been found in BMD patients who remained ambulant until 61 years of age [49]. Introduction of this internally-deleted minigene into dystrophin-deficient *mdx* mice prevented the development of dystrophic pathology [50]. Similar findings have also been reported using a variety of microdystrophin variants in murine and canine DMD models [51–56].

An understanding of the biochemical structure of dystrophin, combined with the genetics of mildly-affected BMD patients, led to the idea that the generation of ‘BMD-like’ internally-deleted dystrophin proteins, which retain partial functionality, could be used to treat DMD. This is the foundational concept underlying microdystrophin gene replacement therapy, antisense oligonucleotide-mediated exon skipping, and many types of CRISPR-Cas9-mediated gene correction therapies for DMD [57]. We have recently reviewed these types of therapies elsewhere [57–59]. Four exon skipping compounds have been approved by the US Food and Drug Administration (FDA) via the accelerated approval pathway; eteplirsen, viltolarsen, golodirsen, and casimersen [57]. In 2023, the first gene replacement therapy for DMD also received FDA approval (Elevidys) [60].

## Adeno-associated virus

Gene replacement therapy aims to treat or manage disease via the delivery of a transgene sequence. Adeno-associated virus (AAV) has emerged as the vector of choice for gene therapy applications, including in the context of DMD [61]. AAV is a non-enveloped virus from the *Dependoparvorius* genus [61]. AAV is often described as non-pathogenic, as it does not cause a specific infection-associated disease in humans, exhibits low immunogenicity, and its replication is dependent on co-infection with a helper virus (such as adenovirus) [62–67].

**Fig. 1:**
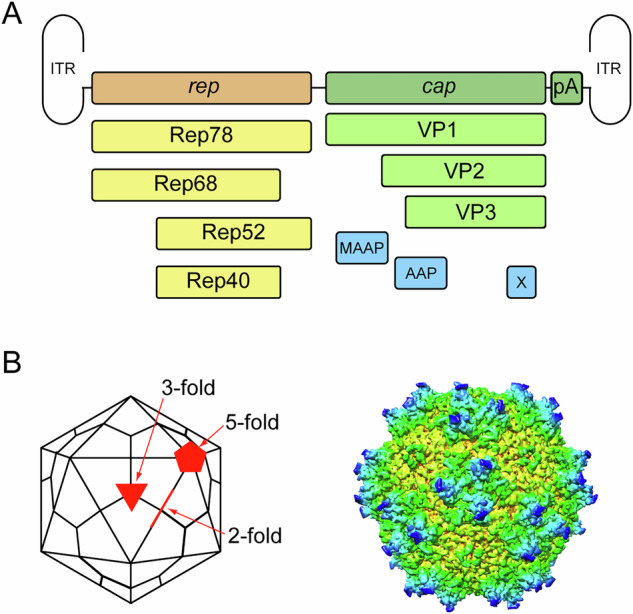
AAV genome and structure. **A** The genome organization of an adeno-associated virus (AAV). The genome contains an inverted terminal repeat (ITR) at each end and two main open reading frames: *rep* (replication proteins) and *cap* (capsid proteins). The *rep* open reading frame encodes four proteins: Rep78, Rep68, Rep52, and Rep40, which are involved in viral replication. The *cap* open treading frame encodes three structural proteins, VP1, VP2, and VP3, which form the viral capsid. Also shown are the accessory proteins assembly-activating protein (AAP), membrane-associated accessory protein (MAAP), and X. **B** A schematic of the icosahedral AAV capsid is shown alongside a crystal structure model of the AAV9 capsid. The 2-, 3-, and 5-fold axes of symmetry are indicated. The crystal structure model was created using Chimera software (UCSF; PDB structure 3UX1) and uses color coding to show the distance of surfaces from the center of the viral capsid.

The AAV genome comprises a 4,680 nt long single-stranded linear DNA molecule (either plus or minus) with 145 nt of palindromic inverted terminal repeat elements (ITRs) at each terminus [68]. It contains two ORFs, *rep* and *cap*. *rep* encodes for four non-structural proteins: Rep78, Rep68, Rep52, and Rep40, which are differentially generated as a consequence of alternative splicing, differential promoter usage, and overlapping reading frames (Fig. 1A) [69]. Rep78 and Rep68 are important for viral DNA replication and site-specific integration into the host genome, while Rep52 and Rep40 facilitate the packaging of the AAV genome into the capsid [69–73].

The AAV capsid (22 nm in diameter) [68] is a highly organized 60-subunit structure with T = 1 icosahedral symmetry. It is composed of VP1, VP2, and VP3 in an approximate ratio of 1:1:10 [74], with all three capsid proteins encoded by the *cap* open reading frame. The entire coding sequence of VP3 is shared among the three proteins, with VP2 harboring approximately 60 additional N-terminal residues, and VP1 containing this region as well as an additional unique N-terminal sequence of ~135 residues. VP3, the smallest of the VP proteins, is 59–61 kDa. Each capsid protein contains a common region (corresponding to VP3) which consists of an 8-stranded β-barrel core (i.e. jelly roll fold) where individual β-strands are connected by flexible surface loop regions, which define the external architecture of the capsid. Both VP1 and VP2 N-terminal extensions contain nuclear localization sequences that aid in nuclear trafficking of the AAV particle [75–77]. Additionally, the VP1 unique N-terminal region harbors a phospholipase A2 (PLA2) domain [28], which mediates endosomal escape by enabling membrane disruption under acidic conditions.

The AAV capsid surface features three distinct symmetry axes: three-, five-, and two-fold (Fig. 1B) [78]. The three-fold axis is characterized by dome-like protrusions that play critical roles in receptor binding and immune recognition, with two significant loops extending above the capsid surface. The five-fold axis of symmetry forms pores, which are hypothesized to facilitate genome packaging and release. A two-fold axis of symmetry is formed by grooves between capsid subunits that contribute to capsid stability.

Three additional proteins are encoded within the *cap* ORF in alternate reading frames: Membrane-Associated Accessory Protein (MAAP) [79], Assembly-activating protein (AAP) [80], and Protein X [81]. Although less studied, these ‘accessory’ proteins are thought to have diverse roles in AAV genome replication, capsid assembly, infection, and host-pathogen interactions.

AAV capsid proteins interact with target cells via the AAV receptor (AAVR, also known as KIAA0319L) and surface proteoglycans (e.g. heparan sulfate) to trigger endocytosis-mediated uptake [82–84]. Co-receptors including integrins, laminins, and growth factor receptors also contribute to AAV internalization [84, 85]. Following viral uptake, synthesis of the second DNA strand is required before viral-encoded genes can be expressed [68]. As such, second strand synthesis constitutes a rate-limiting step in AAV-based gene therapy. AAV vector genomes typically do not integrate into host cell genomic DNA, but instead persist as a circular, episomal head-to-tail concatemers [86, 87], which lowers the risk of insertional mutagenesis [86, 88]. This episomal nature not only enhances safety but also supports long-term transgene expression in postmitotic cells, making AAV an effective tool for treating chronic genetic diseases [89]. However, wild-type AAV can integrate with low efficiency and exhibits a preference for the Adeno-associated virus integration site 1 (AAVS1) region on the q13.4 arm of human chromosome 19, with integration sites on chromosome 5 p13.3 and chromosome 3 p24.3, named AAVS2 and AAVS3 respectively, have also been reported [71, 90]. In contrast, recombinant AAV vectors (rAAV) have been shown to have reduced propensity for integration and with no site-specificity [91].

AAV is capable of transducing both dividing and non-dividing terminally-differentiated cells, highlighting its potential for addressing a plethora of diseases [62, 92, 93]. Multiple AAV serotypes have been isolated and show a broad range of tissue tropism, with AAV1, AAV6, AAV8, and AAV9 being the most promising for targeting skeletal and cardiac muscle [94–97]. The surface variability in VP3 regions contributes to the distinct tropism of AAV serotypes, allowing interactions with specific proteoglycan receptors (such as heparan sulfate for AAV2) [98] and potentially enabling targeted delivery to different cell types.

Importantly, the majority of the AAV genome (96%) is dispensable and can be substituted with a therapeutic transgene to generate recombinant AAV (rAAV) vectors. 4.7 kb is most often reported as the upper limit for exogenous transgene packaging [99], although transgenes with sizes of up to ~5 kb have been packaged into a rAAV [62]. More than 12 AAV natural serotypes have been isolated and a multitude of engineered capsid mutants generated [79, 100]. Additionally, the pool of therapeutically relevant AAVs is not limited to serotypes isolated from humans [101]. For example, AAVrh74, isolated from rhesus macaques, exhibits natural muscle tropism, and is used as the vector system for gene therapy products developed by Sarepta Therapeutics [102]. In addition to naturally occurring serotypes, multiple research efforts are focused on modifying the AAV capsid to improve its muscle targeting. By creating libraries of capsids and selecting for a desired trait, large screening studies can identify capsids with greatly enhanced muscle tropism. Of particular note are the AAVMYO series (1-3) and MyoAAV, both of which were engineered using large parallel screens and show superior ability to target both skeletal and cardiac muscle in vivo [94, 103].

At the time of writing (February 2025), there are six AAV-based products fully approved by the FDA for treatment of hemophilia A (Roctavian) and B (Hemgenix and Beqvez [now withdrawn due to limited market interest]), retinal dystropathy (Luxturna), spinal muscular atrophy (SMA, Zolgensma), and DMD (Elevidys) [104]. Two additional AAV-based therapies were approved in the EU for aromatic L-amino acid decarboxylase deficiency (Upstaza) and familial lipoprotein lipase deficiency (Glybera) [105, 106]. However, Glybera was withdrawn in 2018 due to its high cost and limited demand [107, 108].

The full-length dystrophin ORF is ~11 kb, which exceeds the 4.7 kb packaging capacity of AAV. However, due to the fact that internally-deleted dystrophin protein can be functional (as in case of BMD) numerous shortened dystrophin minigene constructs packageable into an AAV vector have been developed [57]. Current iterations of these constructs are typically referred to as microdystrophins, of which there are multiple variants with distinct constituent domains. Such an approach is in theory applicable to all DMD patients regardless of pathogenic variant type, in contrast with other exon-targeted therapeutic strategies such as exon skipping and CRISPR-Cas9-mediated gene correction [58]. Importantly, microdystrophins are not expected to fully compensate for the loss of dystrophin, given their highly compact, internally-deleted nature, with consequences for their durability and therapeutic efficacy.

## Elevidys: the first AAV microdystrophin gene replacement therapy for DMD

**Fig. 2:**
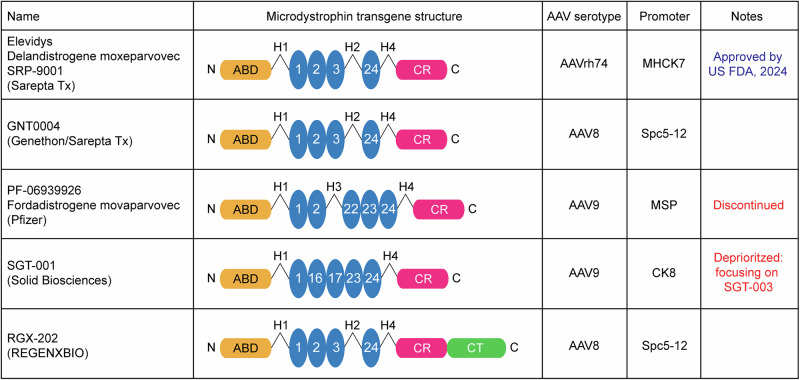
Summary of major microdystrophin therapies. Adapted from Roberts et al., 2023 [57].

In June 2023, delandistrogene moxeparvovec-rokl (Elevidys, previously known as SRP-9001), a microdystrophin gene therapy developed by Sarepta Therapeutics, became the first gene therapy product for DMD to be granted accelerated approval for the treatment of non-ambulatory DMD patients aged 4–6 years by the FDA [60]. Elevidys consists of a codon-optimized microdystrophin transgene lacking spectrin-like repeat domains R4-R23 and the CT domain (Fig. 2). Transgene expression is driven by MHCK7, a synthetic hybrid promoter designed for high expression in skeletal and cardiac muscle tissue that consists of the α-myosin heavy chain enhancer fused to the enhancer, promoter, and a fragment of 5ʹ untranslated region of the muscle creatine kinase gene [109, 110]. Delivery of this microdystrophin transgene is achieved using the muscle-tropic AAVrh74 vector [102] for which the seroprevalence of neutralizing antibodies in DMD patients is low [111]. Approval was granted based on the observed increase in microdystrophin expression in clinical studies, which was considered to be a sufficient surrogate endpoint ‘likely to predict clinical benefits in patients between 4 and 5 years of age’ [112, 113]. In June 2024, approval was expanded to full (traditional) approval for ambulatory individuals 4 years and older, and accelerated approval for non-ambulatory individuals 4 years and older, despite failure to meet primary endpoints in the various clinical trials (described below) [114, 115].

In the first phase 1/2a non-randomized clinical trial of Elevidys in 4 ambulatory DMD patients (NCT03375164), mean microdystrophin expression reached 95.8% of wild-type dystrophin levels, as determined by western blot (adjusted for fibrotic and fatty tissue) at 12-weeks post-injection with no adverse events observed [116]. Although the trial did not include the placebo group as a comparator, improvement in motor scores (i.e. North Star Ambulatory Assessment, NSAA) and reductions in serum creatine kinase (CK) levels were reported in all enrolled patients [116]. These results were sustained up to four years post treatment [117].

A further phase 1/2 randomized, placebo-controlled crossover trial (NCT03769116, *n* = 41 total) reported microdystrophin expression levels which reached a more modest 23.8% of healthy levels 12 weeks post-treatment, and that the treatment was safe for up to two years after dosing [118]. Interim data from a phase 1 open-label trial evaluating long-term safety and efficacy of Elevidys (up to 3 years, NCT04626674, ENDEAVOR) reported microdystrophin expression levels of 54.2% of healthy levels 12 weeks post treatment, together with immunofluorescence staining showing correct localization of dystrophin protein at the sarcolemma [119]. Notably, two, new treatment-related serious adverse events were reported in the ENDEAVOR study: myocarditis and immune-mediated myositis [119].

A subsequent randomized, placebo-controlled phase 3 trial of Elevidys failed to meet the primary end-point of a change in the NSAA score at week 52 post-infusion (NCT05096221, EMBARK, *n* = 125 total) [120]. Analysis of secondary endpoints showed numerical improvements favoring treatment, although no statistical significance was claimed [120]. However, such analyses were discussed further in the memorandum document explaining the FDA decision to grant full approval for Elevidys. Specifically, the justification for the approval decision was based on statistically significant improvements in the secondary endpoint measures time to rise from the floor (TTR) and 10 m walk/run test [121]. Furthermore, four-stair climb (Ascend4) performance and serum creatine kinase levels also statistically improved in Elevidys-treated individuals compared to the placebo group [121]. These improvements are considered clinically meaningful based on minimal clinical important difference values [122]. It was noted that TTR and Ascend4 have themselves been utilized as primary endpoints on which the DMD drugs Vamorolone and Givinostat were approved, respectively [121]. Notably, the decision to approve Elevidys was not supported by the FDA statistical reviewers (https://www.fda.gov/media/179489/download).

Further Elevidys clinical trials are ongoing; ENVISION (NCT05881408) and EXPEDITION (NCT05967351). Notably, the EXPEDITION study aims to monitor safety and efficacy of a large number of patients treated with Elevidys in previous clinical trials. The minimum follow-up time is five years post infusion with the study completion date estimated towards the end of 2030. In March and June 2025, Sarepta reported that two non-ambulatory DMD individuals treated with Elevidys had died of acute liver failure [123, 124], although further details are not publicly available at the time of writing. As a result of these events, Sarepta has suspended shipments of Elevidys for treatment of non-ambulatory DMD patients and paused the ENVISION trial (NCT05881408) while an enhanced immunosuppressive regimen for non-ambulatory patients is evaluated.

## Other microdystrophin gene therapy clinical programmes

There are five other notable competitor microdystrophin drugs that have been assessed in clinical trials sponsored by Pfizer, Solid Biosciences, Genethon (in partnership with Sarepta), REGENXBIO, and Insmed. These gene therapies differ in terms of (i) the structure of their microdystrophin payloads, (ii) the choice of promoter, and (iii) the AAV serotypes (Fig. 2). Typically, these gene therapies are administered via the intravenous (IV) route, with the exception being INS1201 (Insmed, NCT06817382), which is intrathecally injected. Microdystrophin clinical trials are summarized in Table 1. After Elevidys, the gene therapy developed by Pfizer, fordadistrogene movaparvovec (PF-06939926), is the most extensively studied, although Pfizer has recently moved to discontinue its development. PF-06939926 is an AAV9 vector encoding a microdystrophin transgene driven by a muscle-specific promoter [57, 125]. Safety and efficacy of PF-06939926 were investigated in a phase 1/2 clinical trial (NCT03362502) in 23 ambulatory and non-ambulatory DMD patients. Initial results in 6 treated boys demonstrated that the treatment was well tolerated. However, one boy developed acute kidney injury accompanied by hemolysis and reduced platelet count. These symptoms resolved upon hospitalization and treatment, with the participant’s renal function returning to normal within 15-days post-treatment [126].

**Table 1:** Summary of microdystrophin gene therapy clinical trials.

			Dysrophin		Endpoint		SAEs
Study Number/NAME (Phase)	Sample Size (age, years)	Dose (vg/kg)	% Positive Fibers	% Protein by Western blot	Primary endpoint outcome	Secondary endpoint outcome	
Sarepta Tx – Elevidys (also known as Delandistrogene moxeparvovec-rokl, SRP-9001)							
NCT03375164 (1/2)	4 (4–7)	2×10^14^	81.2% at 12 weeks (mean)	74.3% (mean)	TRAEs: 18 TRAEs were observed. All were considered mild or moderate and resolved.	NSAA change of +7.0 (SD = 2.9) four years post-treatment	None reported
NCT03769116 (1/2)	41 (4–7)	2×10^14^	CFBL: 23.82% - 39.64% at week 12 (mean)	CFBL: 23.88% - 78.92% of normal at week 12 (mean)	Change in microdystrophin expression from baseline, NSAA increase of 1.7 (SD = 0.6) at week 48	Change from baseline in time to rise from floor: −0.15 (SD = 0.25)	Liver injury, hypertransaminas-aemia and rhabdomyolysis
NCT04626674 ENDEAVOR (1b)	55 (2 + )	1.33×10^14^	CFBL: 54.2% (SD = 42.6) at week 12 (mean)	CFBL: 48.3% (SD = 25.4) of normal at week 12 (mean)	CFBL of microdystrophin expression	Safety: 177 TEAEs observed in 19 patients, most mild of moderate	Immune-mediated myositis, myocarditis, increased transaminases
NCT05096221 EMBARK (3)	126 (4–7)	1.33×10^14^	34.29% at week 12 (mean)	Not reported	CFBL in NSAA (week 52) - not met	Change in microdystrophin expression, time to rise from floor: −0.27 s (SD = −0.56)	Myocarditis, left ventricular dysfunction, gamma-glutamyl transferase and transaminase increased, rhabdomyolysis
NCT05881408 ENVISION (3)	148 (4–7)	1.33×10^14^	Not reported	Not reported	CFBL in the Total Score of PUL (Week 72) – not reported	Microdystrophin expression and functional outcomes - not reported	Not reported
NCT05967351 EXPEDITION (3)	400 (4–7, follow-up study)	NA	Not reported	Not reported	Safety up to 5 years post treatment – Not reported	CFBL in NSAA (5 years post treatment) – Not reported	Not reported
NCT06128564 (2)	21 (4–7)	1.33×10^14^	Not reported	Not reported	Number of TRAEs, SAE and AESI up to week 260 – Not reported	Change in microdystrophin expression by WB – Not reported	Not reported
NCT06241950 (1)	6 (4-9)	1.33×10^14^	Not reported	Not reported	CFBL of microdystrophin expression by WB and IF, vector genome copies in muscle tissue biopsy – Not reported	Cmax of Imlifidase, IgG in serum and rAAVrh74 Antibody Titers after Imlifidase administration, vector genome copies, safety – Not reported	Not reported
NCT06270719 ENDURE (4, observational)	500 (4 + )	NA	Not reported	Not reported	CFBL in 10mWT at month 12 – Not reported	Functional and patient reported outcomes – Not reported	Not reported
NCT06597656 HORIZON (1)	16 (4–8)	1.33×10^14^	Not reported	Not reported	CFBL of microdystrophin expression by WB and IF, vector genome copies in muscle tissue biopsy – Not reported	TEAEs, SAEs and AESI, change from baseline in rAAVrh74 antibody titerss – Not reported	Not reported
Genethon/Sarepta Tx – GNT0004							
GNT-016-MYDF EUDRACT: 2020-002093-27 (1,2,3)	5 (6–10)	1×10^13^–3×10^13^	15–85%	Not reported	4.7 improvement in NSAA score at 1 year. Further improvement or stabilization at 2 years	Safety, PK/PD – Not reported	None reported
Pfizer – PF-06939926 (also known as Fordadistrogene movaparvovec)							
NCT03362502 (1)	19 (2-3)	1×10^14^– 3×10^14^	38–69%	Not reported	Safety, clinical evaluation – SAEs reported	Minidystrophin expression, safety up to 5 years post treatment – Not reported	Dehydration, acute kidney injury, thrombocytopenia. One patient died.
NCT04281485 CIFFREO (3)	122 (4–7)		Not reported	Not reported	NSAA change at 52 weeks – not met	Minidystrophin expression by LC-MS and IF, CFBL in serum CK, functional outcomes – not reported	None reported
NCT05429372 DAYLIGHT (2)	10 (2-3)	2×10^14^	Not reported	Not reported	Safety and tolerability	Minidystrophin expression by LC-MS and IF, safety and tolerability – Not reported	One patient died over one year after infusion (cardiac arrest)
Solid Biosciences – SGT-001 or SGT-003							
NCT03368742 IGNITE DMD (1/2)	12 (4-17)	5×10^13^–2×10^14^	1–70% at week 12	BLQ – 17.5% at week 12	Safety and tolerability – most common TEAEs: nausea, emesis, pyrexia, thrombocytopenia, and headache	Safety and efficacy measured by microdystrophin expression and functional outcomes (NSAA, 6MWT, PUL, cardiac function and quality of life scores) – continued improvements up to 1.5 years after treatment	Complement activation within first weeks following dosing (3 patients)
NCT06138639 INSPIRE DUCHENNE (1/2)	43 (4-11)	1×10^14^	78% at day 90 in 3 patients (mean)	110% at day 90 in 3 patients (mean)	Safety and tolerability – most common TRAEs: nausea and vomiting, transient thrombocytopenia, infusion related hypersensitivity reaction, and fever	CFBL in microdystrophin expression, NSAA and SV95C - Not reported	None reported
REGENXBIO – RGX-202							
NCT05693142 AFFINITY DUCHENNE (2/3)	65 (1 + )	1×10^14^ or 2×10^14^	39.7% - 120%	Not reported	Safety and tolerability (part 1), pharmacodynamics (part 2 and 3) – Not reported	Efficacy measured by functional outcomes – improvement across variety of scales compared to natural history data – Not reported	None reported
NCT06491927 (Observational)	~19	1×10^14^ or 2×10^14^	NA	NA	Long term safety – Not reported	Efficacy measured by change in functional outcomes – Not reported	Not reported
Insmed – INS1201							
NCT06817382 ASCEND (1)	~12	Not disclosed	Not reported	Not reported	Incidence and Severity of TRAEs up to week 96 – Not reported	Dose finding, change from baseline in microdystrophin RNA and protein at weeks 16 and 48 – Not reported	Not reprorted

*AESI* adverse event of special interest, *BLQ* below limit of quantification, *CFBL* change from baseline, *NSAA* north star ambulatory assessment, *PUL* performance of upper limb, *SAE* serious adverse event, *TRAE* treatment-related adverse event, *TEAE* treatment-emergent adverse event, *10mWT* 10 m walk/run test. *6MWT* 6 min walk test. ‘Not reported’ means no data publicly available at the time of writing. ‘None reported’ means data is available and no SAEs were reported. ‘Not disclosed’ means known to sponsor, but not disclosed to the public at the time of writing. ‘NA’ means not applicable.

Expression of microdystrophin after PF-06939926 administration was confirmed by immunofluorescence staining at week 8 post-treatment. Muscle biopsies taken from the biceps showed a mean of 69% positive fibers in participants receiving the higher 3 × 10^14^ vg/kg dose, and a mean microdystrophin protein level of ~30% of normal was measured by liquid chromatography mass spectrometry [126]. Transgene expression was sustained for 1-year post treatment [127]. Pfizer also reported improvement in the NSAA scores at 1-year post-treatment for nineteen ambulatory patients receiving the therapy [128]. Based on these results, PF-06939926 received a fast-track designation from the FDA [127]. Further data from the NCT03362502 study reported in March 2021 demonstrated both efficacy and an acceptable safety profile [129]. At that stage, the study was extended to non-ambulatory DMD patients, while Pfizer reported three serious adverse events (acute kidney injury, thrombocytopenia, and dehydration) which resolved within three weeks post-dosing [129]. In November 2020, a phase 3 study evaluating safety and efficacy of PF-06939926 was initiated (NCT04281485, CIFFREO). It included 122 ambulatory DMD patients, with a primary aim to assess the efficacy of PF-06939926 based on the change from baseline of NSAA scores one-year post dosing. The study included follow-up for up to five years post treatment for all patients [130]. Notably, in September 2021 three serious, treatment-related adverse events of muscle weakness including myocarditis were reported in NCT04281485, leading to an amendment of the study protocol [131]. Notably, these serious adverse events resembled effects observed in both the ENDEAVOR study led by Sarepta [119] and in another trial investigating the safety of a distinct microdystrophin construct (GNT0004) developed by Genethon (Eudra-CT number, 2020-002093-27) [132].

These serious side effects of microdystrophin gene therapy across clinical trials prompted a collaborative investigation into the underlying cause. In a situation perhaps unique to DMD, a working group chaired by academic experts was established that enabled pharmaceutical companies/sponsors (Sarepta, Pfizer, Solid Biosciences, and Genethon) developing microdystrophin gene therapies to share data concerning severe adverse reactions across sponsor clinical programmes [132]. This effort established that affected patients had common pathogenic variants in the *DMD* exon 8–11 region (encoding the H1 hinge, which is important for force production) [133], such that they were naïve to certain transgene epitopes that were supplied in the gene therapy product. As such, clinical trial inclusion criteria were subsequently adjusted to exclude those patients carrying large *DMD* deletions which correspond to equivalent transgene sequences, in an effort to minimize anti-transgene immune responses [132]. This example provides a model for cooperation between industry and academics for future drug development efforts. However, Pfizer later reported the unexpected deaths of two DMD clinical trial participants: in December 2021 (NCT03362502) [134] and in May 2024 (NCT05429372, DAYLIGHT) [135]. Around the same time, the company reported that the ongoing CIFFREO phase 3 trial had failed to meet either the primary endpoint of an improvement in NSAA score or other motor function scores one year post dosing [136]. In July 2024, Pfizer announced the discontinuation of its PF-06939926 development programme (Table 1) [137].

## Considerations for effective AAV microdystrophin gene therapy

### Anti-transgene and anti-AAV immune responses

As described above, anti-transgene immune responses constitute an important barrier to effective AAV microdystrophin gene therapy. Myofibers are capable of presenting antigens to CD4+ and CD8+ T cells via incompletely understood pathways [138–140]. Due to the continuous muscle degeneration and regeneration in dystrophic tissue, the permeability of the sarcolemma is enhanced, which may facilitate the leakage of microdystrophin-derived antigens from the myofibers, as has been observed for reporter transgenes [141]. These neoantigens can activate patrolling immune cells such as dendritic cells or macrophages, which could elicit both innate and adaptive immune responses against the transgene protein or viral capsid, leading to decreased therapeutic effectiveness or immunotoxicity [142, 143].

A further obstacle to effective microdystrophin gene therapy is the relatively high prevalence of neutralizing antibodies (NAbs) against AAV in the general population due to previous exposure to wildtype virus. NAbs can opsonize AAV, preventing it from entering the host cell via its cellular receptor, impairing viral function in the cytoplasm and nucleus [144], and thus reducing overall transduction efficiency [62].

Furthermore, non-neutralizing antibodies can also bind to AAV extracellularly, enhancing its removal via the spleen and leading to lower transduction rates [145]. NAbs against AAV2 are the most prevalent type among 10 different countries [146], with geographical differences having been noted (e.g. prevalence of NAbs against AAV1 being 32–67% in the USA, 48% in Sweden, and 79% in the combined Polish and Hungarian populations) [147, 148]. A study of NAbs against AAV9 in 100 Chinese DMD patients found that 42% were positive, with young patients ( < 4 years) exhibiting low seropositivity rates [149]. Another study in 281 Chinese DMD/BMD patients, found anti-AAV2 NAbs in 66.9% of individuals and anti-AAV9 NAbs in 32.4% of individuals [150]. A separate study found that the seroprevalence of antibodies against AAV9 (36%) and AAVrh74 (32%) was lower than for AAV2 (56%) and AAV8 (47%) [151]. Long-term (6 years) follow-up of DMD patient NAb seropositivity rates showed no change in antibody titres, suggesting that seroconversion is rare [152]. Additionally, cross-reactivity of NAbs between AAV serotypes has been reported [153]. Many clinical trials using AAV gene therapy exclude patients with certain levels of Nabs [154]. However, assays to test for the presence/titer of NAb in patient serum can exhibit high variability [154]. Solid Biosciences, Pfizer, Insmed, and Sarepta Therapeutics have all adopted exclusion criteria based on serum anti-AAV NAb titers exceeding protocol-specific thresholds, and/or if participants have had previous exposure to DMD gene therapy products (NCT03368742, NCT05096221, NCT04281485, NCT03375164, NCT06817382). Similarly, the presence of anti-AAV8 antibodies is an exclusion criterion for both Genethon [155] and REGENXBIO (NCT05693142) microdystrophin clinical trials.

AAV-mediated complement activation presents an additional source of inflammation. In vitro studies by Zaiss et al. demonstrated that AAV capsid proteins interact with proteins of the complement cascade, such as iC3b or complement regulatory protein H [156]. Complement activation towards AAV gene therapies can lead to thrombocytopenia and thrombotic microangiopathy, which are serious adverse events that required careful monitoring [126, 156–160]. In the case of microdystrophin gene therapy, complement activation and atypical hemolytic uremic syndrome were reported in two clinical trial participants treated with the Pfizer microdystrophin (PF-06939926, NCT03362502). Similarly, thrombocytopenia was also reported as a serious adverse event in response to treatment with the Solid Biosciences microdystrophin gene therapy (SGT-001, NCT03368742) [107].

While immune responses to vector and transgene poses a significant challenge to AAV gene replacement therapy, several actions can be taken to modulate the immune system prior to treatment. Treatment with glucocorticoids (e.g. prednisolone, deflazacort) is the standard-of-care for DMD [161], and has been shown to delay the time to loss of ambulation [162, 163]. Clinical trials for microdystrophin gene therapy have frequently required that participants be on a stable steroid regime for a defined period prior to participation. For example, the ENDEAVOR trial included a requirement for a stable dose of oral glucocorticoids for at least 12 weeks before screening in certain cohorts (although steroid-naïve cohorts were also included in this trial design) (NCT04626674). Furthermore, it is also common that trial participants are placed on additional ‘top-up’ steroid regimes prior to infusion and continued for a period after treatment to attenuate immune responses directed towards the therapy [164, 165]. For example, an additional 1 mg/kg/day (prednisone equivalent) was administered the day before infusion and maintained for ≥60 days post-AAV exposure in the ENDEAVOR trial [165]. Importantly, given the beneficial effects of steroid treatment on delaying DMD progression, it has been noted that the increased dose steroid regimes co-administered with gene therapy are an important potential confounding variable that should be considered when assessing the efficacy of microdystrophin gene therapies [165].

Transient immune suppression is thought to provide time for AAV clearance, causing less anti-capsid protein-mediated CD8+ T cell activation [166]. The most commonly used immunosuppressive drug used for AAV gene therapy is prednisolone [164], although other regimens including anti-thymocyte globulin, cyclosporine, mycophenolate mofetil, and tacrolimus have also been used [164, 167–172]. In vivo studies by Wang et al., demonstrated that use of these immunosuppressants in *cxmd* (canine X-linked muscular dystrophy) dogs treated with AAV-microdystrophin therapy, could reduce T cell activation and enhance long-term transgene expression [168]. Other approaches to reduce the levels of NAbs include inhibiting their production by treatment with rituximab (anti-CD20 monoclonal antibody) or the combination of sirolimus (rapamycin) and prednisolone, and plasmapheresis to eliminate circulating NAbs [173–176].

Interestingly, mesenchymal stromal cell (MSC) infusions could present a cell-based approach to mitigate the immune response and achieve long term transgene expression. To this end, infusions of MSCs were performed in one dystrophic CXMD_J_ dog prior to administration of AAV9-microdystrophin [177]. The authors reported reduced IFN-γ levels in peripheral blood mononuclear cells re-stimulated with AAV9 ex vivo, indicating that the MSC pre-treatment blunted the immune response to subsequently injected AAV9. Importantly, the dog receiving MSCs prior to AAV9-microdystrophin showed higher expression of the transgene and more improvement in functional measures, such as gait, muscle atrophy, and dysphagia. The authors theorize that pretreatment with MSCs could enable lower doses of AAV gene therapies while preserving efficacy [177].

Capsid engineering could aid not only NAb evasion but also enable repeated administration of vectors, which could improve the efficacy of gene therapy applications. The rAAV2.15 and 2.4 vectors were designed to evade NAbs in vitro and in vivo in mice without affecting the tropism [178]. Even though some newly-engineered AAV variants (e.g. MyoAAV1A and 2 A) still cause NAb responses [103], their increased muscle tropism might enable lower dosing regimens, and a favorable immunologic profile with less complement activation and thrombocytopenia [179].

### AAV transduction of muscle stem cells

Skeletal muscle growth and regeneration is supported by a pool of muscle-resident stem cells called satellite cells [180]. In healthy adult tissues, these mononuclear cells are kept in a reversible quiescent state at the periphery of skeletal muscle myofibers until they become activated upon injury, expand, undergo myogenic differentiation, and subsequently fuse to form multinucleated myotubes (and eventually mature myofibers) [181]. Transduction of satellite cells is desirable, as they will contribute additional dystrophin-expressing myonuclei to myofibers, leading to an accumulation of dystrophin protein throughout the life of the treated individual, provided that AAV episomes are not lost or diluted as a consequence of cell division. Notably, there is conflicting evidence as to whether AAV can transduce satellite cells. Arnett et al., have reported that satellite cells are refractory to transduction after intramuscular injection of AAV6 or AAV9, while treatment with AAV8 resulted in transduction of fewer than 5% of satellite cells [182]. Conversely, systemic injection with AAV9 vectors carrying Cas9 and sgRNA expression cassettes was shown to result in gene editing in satellite cells [183, 184]. The development of AAV vectors with improved capabilities for transducing muscle satellite cells therefore has the potential to greatly enhance the therapeutic effectiveness of microdystrophin gene therapies.

Interestingly, Dumont et al. have reported that, in healthy satellite cells, dystrophin binds to the cell polarity regulator MARK2 and thus contributes to correct asymmetric cell division and maintenance of the pool of myogenic progenitors within the skeletal muscle [185]. Notably, dystrophin interacts with MARK2 via R8 and R9 repeats [186], which are absent from all the microdystrophin variants approved or currently under investigation in clinical trials. Improved microdystrophin variants in which the MARK2 interaction is preserved may be needed to correct the intrinsic satellite cell defect in DMD.

### Transgene persistence

As described above, the vast majority of transgenes persist as an episomal, circular duplex concatemer in transduced cells following administration, with very limited evidence of chromosomal integration [187]. In spite of this, animal studies have shown that transgene persistence and long-term expression over months and years is feasible [188, 189]. A study in a canine hemophilia model reported transgene expression for more than eight years after a single gene therapy administration [188]. Another study by Nathwani et al. found a dose-dependent correlation between long-term transgene expression and the administered AAV dose [189]. They reported >10% of normal expression levels over five years in nonhuman primates after a single IV AAV injection delivering a human codon optimized coagulation factor IX (hF.IX). However, a steady reduction in transgene number, transgene expression, and transduced cells was observed [189].

The extent to which poor transgene persistence will complicate microdystrophin gene therapy is unclear. Mature myofibers are terminally-differentiated and non-dividing syncytial cells, and so are therefore not subject to vector genome dilution that may occur in other tissues composed of dividing single-nucleated cells. However, the absence of functional dystrophin protein in DMD muscle results in chronic cycles of myonecrosis and compensatory regeneration and therefore substantial muscle turnover [190]. As such, episomal AAV vector genomes will likely be progressively lost from dystrophic muscle over time [143]. Indeed, this was the case in a study examining AAV-delivered microdystrophin in the *mdx* dystrophic mouse model, with dystrophin expression detected in 35–50% of myofibers after two months but only in 20–30% after 4 months [191]. However, other studies of systemic microdystrophin delivery in the same mouse model have shown impressive durations of transgene expression in skeletal and/or cardiac muscle up to 18 months (i.e. lifelong) after systemic injection [52, 192–195]. Notably, the degree of degeneration and regeneration in the *mdx* mouse is substantially less than observed in DMD patient muscle (thereby underestimating the degree of vector genome loss that is likely in the human situation), which limits the translatability of these findings. Additionally, canine studies showed that microdystrophin delivered by an AAV9 vector was detected in skeletal and cardiac muscle after six months, with some muscles demonstrating expression in more than 75% of myofibers [196].

Other factors may also contribute to a decline in AAV-encoded transgene expression. For example, it has been proposed that double-stranded RNAs derived from the AAV ITR can be recognized by cellular pattern recognition sensors (e.g. RIG-I and MDA5), leading to interferon induction that ultimately diminishes transgene expression [197]. Similarly, vector DNA may become epigenetically silenced over time, leading to a reduction in transgene expression [198]. The dystrophic muscle environment itself can also constitute an obstacle to transgene expression and persistent therapeutic effect. Along these lines, Mollard et al., have reported that the post-regeneration environment is inherently resistant to AAV1-encoded transgene expression in mice [199], and direct damage to transgene-derived transcripts by reactive oxygen species in dystrophic muscle has also been described [200].

In the context of DMD, long-term microdystrophin expression was seen in *mdx* mice after IV delivery of microdystrophin (Elevidys) at a dose of 6×10^12^ vg [52]. Six months after therapy, 65% microdystrophin positive fibers were observed and muscle force was increased compared to baseline [52]. Moreover, a study by Le Guiner et al., achieved long-term microdystrophin expression and an improvement in muscle function over two years in non-immunosuppressed golden retriever muscular dystrophy (GRMD) dogs [54]. In this study, IV administration of rAAV2/8.Spc5-12, which harbors a codon-optimized canine microdystrophin (ΔR4-23/ΔCT), did not induce an adaptive immune response, potentially due to the nature of the transgene or the synthetic promoter sequence [54].

Recently, it was shown in D2.*mdx* mice (a severe DMD model) [201] that treatment with certain different microdystrophin constructs resulted in an acceleration of cardiac pathology, which was attributed to competition between the microdystrophin transgene product and utrophin at the cardiomyocyte membrane [202]. This effect was not observed for all microdystrophin variants, suggesting that small alterations in transgene design may have major differences in functional outcomes.

## Discussion

The approval of Elevidys as the first gene therapy for DMD is a landmark event in the development of therapeutics for DMD. This review has focused on this drug, given its recent approval, and because it is the microdystrophin therapy for which the most peer-reviewed information is available. By contrast academic literature is relatively scarce for the other microdystrophin gene therapy programmes. Importantly, the efficacy of Elevidys is debatable, with a failure to reach a primary endpoint at phase 3 [120]. Development of the now discontinued competitor drug PF-06939926 was overshadowed by the deaths of two clinical trial participants. During the preparation of this manuscript, Sarepta also reported the death of two DMD patients with acute liver failure following treatment with Elevidys [123, 124]. At the time of writing, Sarepta has agreed to temporarily pause shipments of Elevidys at the request of the FDA. Notably, other trial participant deaths after AAV therapy have been reported in trials for X-linked myotubular myopathy [203], mucopolysaccharidosis type III [204], and for two patients treated with Zolgensma, an approved AAV gene therapy product for SMA [205]. Furthermore, an adult DMD patient died eight days after treatment with an AAV-delivered CRISPR therapy, with a vector-associated innate immune reaction leading to acute respiratory distress and cardiac arrest [206]. With the exit of Pfizer from this space, it will be interesting to see how the competitor gene therapy products fare in the near future. Solid Biosciences has also recently announced that it is pausing development of its microdystrophin therapy SGT-001 in order to focus development on its next generation gene therapy product, SGT-003, which utilizes a novel, muscle-tropic AAV capsid (AAV-SLB101) that has been rationally designed to target integrin receptors in order to promote transduction of skeletal and cardiac muscle, while decreasing liver targeting [207, 208]. Importantly, the SGT-001 and SGT-003 microdystrophins include the R16/17 repeats, which are important for anchoring nNOS at the sarcolemma, a degree of functionality that may be lacking with other microdystrophin designs. Initial data from the phase 1/2 INSPIRE trial (NCT06138639) in 6 DMD patients is highly encouraging, with no safety issues and a mean of 110% microdystrophin levels at 90 days post treatment in the first three DMD boys dosed [208]. Promising improvements in some metrics of cardiac functions were also observed in some trial participants [208].

Furthermore, the approval of Elevidys may also impact the use of approved exon skipping compounds, especially if stakeholders favor a one-time gene therapy over a lifetime of exon skipping treatment. Importantly, the approval of Elevidys (and for that matter, the exon skipping compounds for DMD) is unlikely to be similarly achieved in non-US markets (e.g. European Union) based on the current paucity of clinical benefit demonstration and high cost ($3.2 million per infusion) [209]. As such, there is clearly still room for vector and transgene improvement, as the clinical challenge of treating DMD is far from met.

A key issue in development of dystrophin restoration therapies is the quality of the restored dystrophin. Specifically, microdystrophin minigenes lack large internal regions and may also be truncated at the C-terminus compared to full-length dystrophin (Fig. 2). These proteins are expected to be partially functional, but likely incapable of completely compensating for dystrophin loss. The relative functionality and protein stability (influencing therapeutic durability) is expected to be different among microdystrophin designs, although true head-to-head comparisons of the effectiveness of these therapies in humans will be difficult to perform.

Future challenges include the goal of delivering full-length dystrophin transgenes through advanced exon skipping strategies [210, 211], gene editing [212], split-vector approaches [213–215], or higher packaging capacity vectors [216–218]. Given the safety concerns regarding treatment with high dose AAV [219], there is a need for improved therapies that can provide enhanced efficacy at lower doses. Additionally, a microdystrophin gene therapy approach may be combined with a next generation exon skipping approach for synergistic benefit. Patients treated with a microdystrophin therapy may require a ‘top-up’ later in life, which could be achieved with exon skipping compounds in patients with amenable variants. Such an approach has been investigated in the case of Zolgensma, an AAV gene therapy for SMA. A phase 4 open-label trial (RESPOND, NCT04488133) investigating the use of Spinraza (a splice switching oligonucleotide) in SMA patients who showed minimal improvement after treatment with gene therapy (Zolgensma) has reported interim findings [220]. Patients treated with Spinraza showed reduced levels of plasma neurofilament light chain (a marker of neurodegeneration), and improvements in motor function were observed for most participants over baseline [220]. However, a separate study found that such a dual therapy offered little additional benefit over gene therapy alone in SMA type I infants, despite being well-tolerated [221].

Conversely, pre-treatment with exon skipping compounds prior to administration of AAV may improve the effectiveness of the latter, as transient stabilization of muscle turnover minimizes vector genome loss in animal models [222]. While the efficacy of current exon skipping compounds is low, next generation antisense oligonucleotides conjugated to delivery-assisting moieties like cell penetrating peptides (i.e. peptide-PMO, PPMO) or antibodies may make such approaches more realistic for DMD combination therapies [57, 59].

It will also be important to understand the optimal timing of treatment. Early dosing with Zolgensma for the treatment of SMA has demonstrated a very clear benefit [223]. Whether such an early dosing strategy would be favorable in DMD is currently unknown. An infant treated with an AAV-delivered exon skipping therapy showed a very positive response with ~99% of fibers being dystrophin-positive at biopsy four weeks post-treatment [210, 211, 224, 225]. Whether similar such positive effects can be maintained over time with microdystrophin gene therapy remains to be determined. Indeed, beneficial effects may be counteracted by the loss of vector genes and/or a dilution effect as non-microdystrophin-expressing myonuclei are progressively added to myofibers through the process of growth and repair. A further issue gaining increasing recognition, is the importance of uniformity in dystrophin expression, as the various therapeutic modalities can influence the pattern of dystrophin restoration at the sarcolemma [226–229]. The importance of this issue in relation to microdystrophin therapy has not yet been extensively studied. However, incomplete transduction of myonuclei, vector genome loss or episome silencing, and addition of non-microdystrophin expressing myonuclei all may contribute to the generation of myofiber heterokaryons whereby microdystrophin is expressed in some regions, but not others.

In conclusion, the concept of internally-deleted dystrophin minigene therapies has made the leap from bench to bedside and FDA-approval in the case of Elevidys. Multiple other microdystrophin gene therapy clinical programmes are ongoing, and the challenge of addressing DMD remains. Future developments will likely see improved vectors, and a transition towards therapies which can achieve full-length dystrophin protein expression in the muscles of DMD patients.
